# Changes in farmers' knowledge of maize diversity in highland Guatemala, 1927/37-2004

**DOI:** 10.1186/1746-4269-2-12

**Published:** 2006-03-01

**Authors:** Jacob van Etten

**Affiliations:** 1 Technology and Agrarian Development and Centre for Geo-Information, Wageningen University, Wageningen, The Netherlands

## Abstract

Small-scale studies on long-term change in agricultural knowledge might uncover insights with broader, regional implications. This article evaluates change in farmer knowledge about crop genetic resources in highland Guatemala between 1927/37 and 2004. It concentrates on maize (*Zea mays *ssp. *mays *L.) in one Guatemalan township, Jacaltenango, an area with much ecological and maize diversity. It relies on a particular type of baseline information: lists of farmer-defined cultivars drawn up by ethnographers in the first half of the twentieth century. A questionnaire format based on two independent lists of local farmer cultivars dating from 1927 and 1937 was used to assess changes in maize diversity. Comparisons between attributes given to each cultivar in the past and in 2004 were used as a partial test of the stability of cultivar identity. In farmers' perceptions, cultivar loss was low and limited to certain cultivars adapted to the warmer environments. Crop production problems were mentioned as the main motives for change. No evidence for a loss of cultivars due to the political violence of the 1980s was found. In the lower areas many newly introduced cultivars were found, which reportedly provide solutions for the production problems the older cultivars have. The article contrasts these findings with those of an earlier study which suggested much cultivar loss due to political violence, and draws conclusions about the methodological implications.

## Introduction

The intraspecific genetic diversity of crops in farmers' fields has increasingly received attention due to several convergent social and academic concerns. Crop genetic innovations for and by poor farming households have become an important focus of food security research [1]. Attention is being paid to the role of farmers in supplying seeds, given the limitations of seed supply by the formal sector in poor areas [2]. Since the early 1970s, concerns over the loss of genetic diversity as maintained in traditional agriculture ('genetic erosion') have spurred research as well [3]. Enhancement and protection of crop diversity has also received some international acclaim. The *International Treaty on Plant Genetic Resources for Food and Agriculture *(2004) obliges the signing countries to "promote or support, as appropriate, farmers and local communities' efforts to manage and conserve on-farm their plant genetic resources for food and agriculture."

Monitoring change is central to much research on crop genetic resources. Genetic resources, like other biological resources, are not 'stocks', but ongoing processes. They never remain static and constant energy is spent on maintenance and innovation to secure their reproduction and adaptation. However, long-term change in intraspecific crop diversity is a particularly problematic research subject. To trace change, comparative methods have to be developed, and some type of time series data should be obtained. If change took place over a long period or in the past, research depends on historical information sources of a varying nature and quality.

This study uses one particular type of historical information, which is available for many areas and crops: lists of farmer-named cultivars or crop types. The aim of the research reported here is to bring out some important aspects of changing farmer knowledge related to their perceptions of intraspecific diversity, which are thought to bear on the biological dimensions of crop diversity. It will describe a methodology for dealing with this type of information to study long-term change in farmer cultivar knowledge. The study concentrates on maize (*Zea mays *ssp. *mays *L.) in one township in the highlands of Guatemala where this methodology was applied.

There are several limitations which have to be taken into account when using a comparative approach based on farmer cultivar names. Definitive answers on questions about the relation between cognition and biological reality might be unobtainable as biological information was never collected in the past. In spite of the difficulties of relating cognitive and biological categories directly, it might be argued that approaching the issue from the side of farmer knowledge might give a complementary perspective to the biological one. Farmers' perceptions and knowledge might give privileged insights in the factors that seem most relevant to farmers themselves, and the motivations for choices in crop cultivar management.

Another limitation of this study, which derives from a deliberate methodological choice, is spatial. It concentrated research efforts in one township, thereby limiting itself to a small area. Another study of changes in farmer knowledge of maize cultivar names in the same region has taken a regional perspective [4]. This study will point out the implications of methodological choices of spatial extent and detail. This issue might be relevant to the development of methods in this field of study. In a field of research in which the possibility of manipulating the context is limited, adopting a micro-scale approach might be seen as a form of experimentation, which may produce important new insights [5]. Fine-grained analysis may uncover the hidden meaning of apparent anomalies by interpreting them in the light of a larger system. Small-scale observations may also be relevant to the understanding of a larger system, when they can only be interpreted by indicating the incoherence of the larger system that was thought to be unified. Thus, fine-grained research on cognitive aspects of farmer diversity management might have complementary merits compared to other research approaches. One of the aims of the present paper is to determine what these are.

## Maize diversity and cultivar naming

The present study relied on a survey about farmer knowledge and concentrated on cultivar names. It did not employ biological specimens or photographs, unlike some other studies in this field, and biological diversity was not measured independently in this study area. Thus, the meaning of farmer cultivars as the unit of analysis and the meaning of cultivar names in relation to maize diversity needs some further discussion.

Zimmerer analyzed local changes in crop diversity in terms of *cultivars*, without drawing conclusions about the broader implications of local cultivar losses, because " [t]he basic regional biogeography of cultivars belonging to almost all native crops remains so inadequately understood that the overall significance of change at a local scale cannot be estimated" [6]. (A useful and broadly accepted definition of cultivar is "a variety, strain, or race that has originated and persisted under cultivation or was specifically developed for the purpose of cultivation" [7].) Reservations about the implications of local studies on (farmer-defined) cultivars might be justified in the case of maize, too. To draw out possibilities to link the findings of this study to broader scales and biological units of diversity, a discussion of maize biogeography and the relation between maize genetic diversity and farmer maize classification is needed.

Research on the biogeography of maize in Mesoamerica has mainly revealed coarse patterns of genetic diversity. Maize was probably domesticated in Oaxaca, Mexico, around 7000 B.C. [8]. In Guatemala, like in other parts of the Mesoamerican region, the milpa complex (maize and intercropped species, including different species of beans and squashes) is central to traditional agriculture. The western highlands of Guatemala are an area that harbours some of the highest concentrations of maize diversity worldwide [9,10]. Anderson made an early study of maize in Guatemala, noting the phenotypic purity of Guatemalan maize in comparison with other areas of Latin America [11]. Wellhausen et al. described thirteen races of maize for Guatemala, based on the morphology of the ear, and mapped their geographical distribution in Guatemala [10]. (For a critique of the followed classification methods, see [12].) Hanson, relying on the work of McClintock, Kato and others, indicated that geographic patterns in phylogeny of Guatemalan maize as revealed by chromosome knobs corresponded to a pattern of two-dimensional migration, maize being more related when it was geographically proximate [13]. Also, increased genetic isolation with increasing altitude was evident in this analysis. Bretting et al. describe the isozymatic variation of the identified Guatemalan maize races, and found a broad distinction between lowland and highland races [14].

Although these investigations have examined broad patterns of maize genetic diversity in Guatemala, little is known about genetic patterns in smaller areas. However, ongoing investigations in Mexico might have implications for Guatemalan maize as well. Regional maize research in Oaxaca and Chiapas has demonstrated low marker based differentiation values (F_ST_) between populations (seed lots) and communities [15-17]. These values are interpreted as evidence for considerable gene (seed) flow between farms and communities. Besides, it is pointed out that maize is a cross-pollinating species. Because of crosspollination between adjacent plots, it may be difficult to maintain maize seed lots genetically 'pure' under farmer conditions [18,19].

However, two points qualify the implications of these findings for the present study. First, the precise implications of the cited genetic studies are not entirely clear. The F_ST _values from which the conclusions are drawn should be interpreted cautiously, as the model on which they are based does not discriminate between recurrent gene flow and historical events, including the fragmentation of related subpopulations [20]. The fragmentation of related subpopulations might prove to be important, as in Mesoamerican maize pollen flow between fields and seed mixing have most likely far less impact than seed exchange and replacement, which is frequent and concerns larger numbers of individual plants. Also, the cited studies do not evaluate differentiation of maize with altitude. Meanwhile, field observations suggest that Guatemalan maize populations might prove to show significant geographical structure.

Native maize farmers in Guatemala generally try to preserve purity in observable characteristics, and are thought to be successful in doing so [11,21]. Isolation of broad maize types in different growing areas may contribute to the maintenance of phenotypic and genotypic differences in some highland communities in Guatemala [21,22]. Farmer cultivars of maize in Guatemala are often grown in different places along an altitudinal gradient, and have different characteristics which make their adaptation specific to these places [22-24]. Characteristics important for farmer classification of maize diversity include the length of the growing season, the shape of the cob, and kernel colour and type [22,23,25-30].

The second qualifying point is that even if high levels of gene flow and low levels of differentiation are assumed, the observed phenotypic differences that constitute the possibility of farmer classification of cultivars might still be meaningful. In the cited studies it has been argued that selection of maize seed by Mexican farmers effectively maintains phenotypic differences in ear and kernel characteristics vis-à-vis gene flow [15,16,31]. These phenotypic differences are important for crop production and use. Farmers are observed to strive for maintenance of some ideal crop type in spite of the challenges of gene flow [31]. It has been argued that phenotypic diversity, as an important dimension of genetic diversity, deserves consideration in its own right, in addition to marker-based diversity [16].

Granted that phenotypically distinguished units exist in Mesoamerican maize farming systems, the question remains how cultivar names given by farmers relate to biological units of diversity. It has been established that during several decades a relatively stable classification scheme existed in one Guatemalan highland community [22]. Even so, it was observed in this community that 'new' seed lots introduced from outside the community did not always receive a distinct name, but might be included in existing local categories [also noted by [18]]. Newly introduced cultivars that received a new, distinctive name included a cultivar suited to cultivate recently cleared land for which other cultivars were not suited, and a cultivar that showed to be more adapted to drought than local cultivars. To generalize from these limited observations, it might be stated that incoming seeds will only receive a distinctive name if they are sufficiently different from locally present cultivars or suited to new types of ecological (or other) use.

In any case, farmer cultivar names do not correspond to phenotypic categories in a straightforward way, but their meanings imply additional dimensions important in classification, including their specific use context, occurrence, history, and origin. (This also indicates that the value of visual aids like specimens or photographs during interviews to solve the cultivar identity issue is relative - cultivar classification does not rely on readily observable characteristics only, but is to some degree contextual.)

In a quantitative analysis of maize in Cuzalapa (Jalisco, Mexico), Louette found that seed lots that bore the same cluster name grouped together morphologically [18]. Thus, in spite of the indicated complications, a sufficient degree of association between cultivar names and genetic diversity might be expected to justify a systematic study of cultivar knowledge change as one source of insights into historical change of crop diversity.

## Context and baseline data

Jacaltenango is a Guatemalan township (*municipio*) located in western highlands. The last census (2002) reports 34,397 inhabitants for this township. The majority of inhabitants belongs to the Maya ethnic group and speaks the (main) local language, Popti', while a minority is monolingual Spanish (28%). The area is home to a close wild relative of maize, teosinte (*Zea mays *ssp. *huehuetenangensis *Doebley), first documented in Jacaltenango and its surroundings by Kempton and Popenoe in 1935 [32]. According to Garrison Wilkes, who has monitored the teosinte populations in the region over the last decades, and visited the teosinte populations around Jacaltenango in 2004, this subspecies is risking extinction (G. Wilkes, pers. comm., December 2004).

Several scholars have raised the issue of changing maize cultivars in Jacaltenango. Johannessen observed that especially the large landholders were taking the lead in introducing new maize cultivars into Jacaltenango, and expressed concern about increasing dependence on monetary resources in order to repeatedly buy new 'hybrid' seeds [21]. On the basis of a comparison between Stadelman's [23] data and interviews done in 2001, Steinberg and Taylor concluded that maize diversity knowledge in Jacaltenango and other townships of Huehuetenango seemed to have decreased since 1937 [4]. They indicate that the political violence of the 1980s and its consequences might have contributed to the loss of agricultural knowledge and biodiversity. The present study evaluates these views for Jacaltenango.

Among the literature on the social aspects of life in Jacaltenango, Casaverde's ethnography, which focuses on social organization, was found particularly useful. It suggests a complex ethnic, territorial and social organization in Jacaltenango [33]. The township was affected by political bloodshed during the armed conflict, which formally ended in 1996. Victor Montejo's well-known book *Testimony *is an eyewitness account of political violence in a community of Jacaltenango [34]. For Jacaltenango, the Comisión de Esclarecimiento Histórico reports 46 cases of human rights violations and violent acts between 1980 and 1985, which involved more than 105 killed and disappeared persons [[35], Annex II]. Many people fled from the area, often to Mexico, but others decided to stay or were compelled to do so, often as members of the paramilitary self-defence patrols.

**Figure 1:**
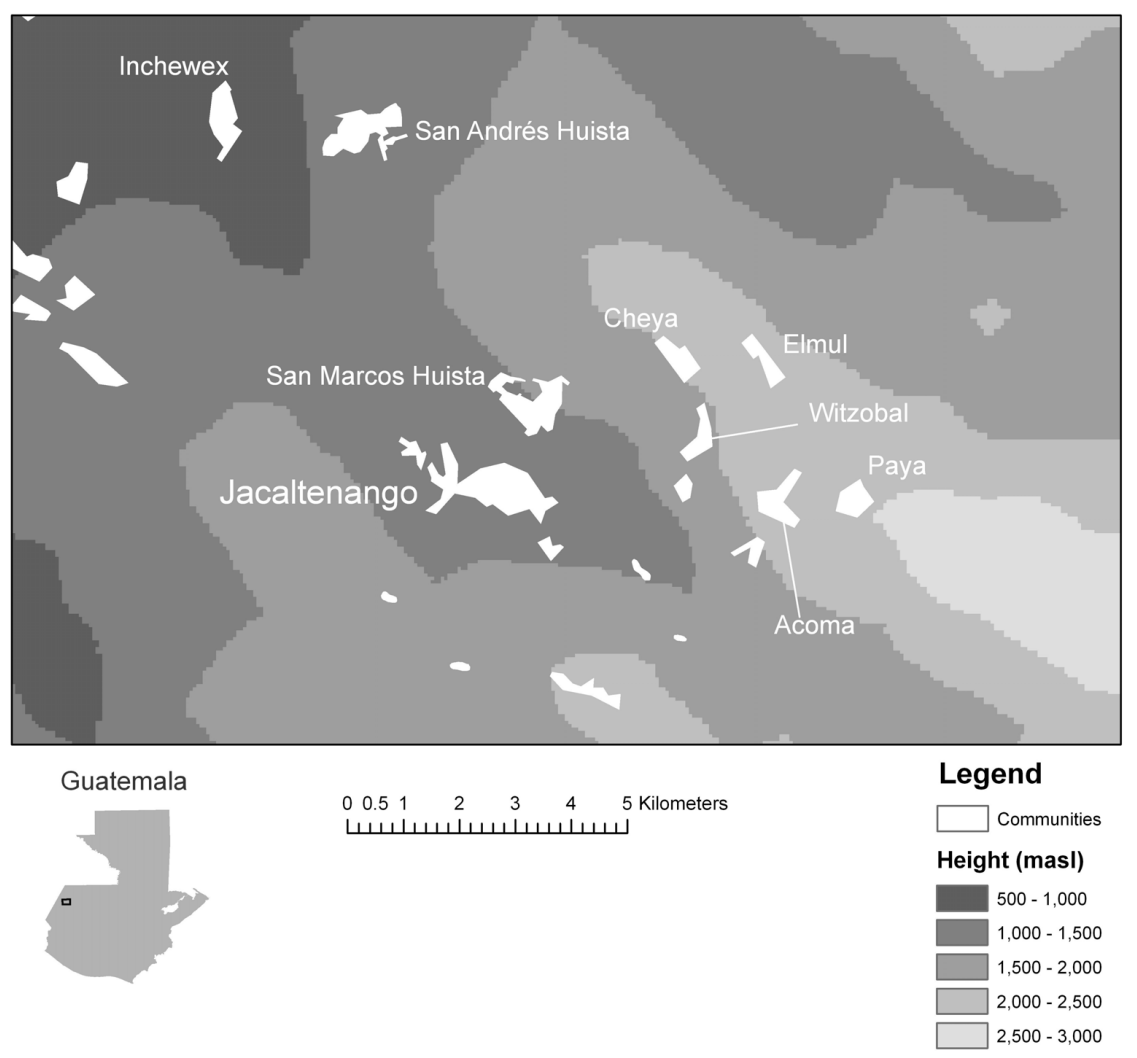
Study area: township capital and eight rural communities of Jacaltenango

The township of Jacaltenango was chosen as a study site for two reasons. First, the number of cultivar names reported in Jacaltenango is the highest for any township in the region [23]. This indicates the exceptional diversity of maize in this township, and is probably related to the fact that the township territory covers an altitudinal transect (Figure 1). Informants usually distinguished three environments: hot (below 1,400 masl) temperate (between 1,400 masl and 2,000 masl) and cold (above 2,000 masl). (The numbers are indicative only; classification is not very crisp.) Second, there was a unique opportunity to study historical change with the availability of two independent cultivar lists made up in the first half of the twentieth century by visiting ethnographers.

In 1927, the township of Jacaltenango was studied by two US ethnographers, Oliver LaFarge and Douglas Byers [36]. In the resulting monograph on traditional Indian culture in the township, the authors mention the remarkable number of farmer maize cultivars in Jacaltenango and give a list of thirteen cultivars and some of their characteristics. In 1937, farmers' knowledge of maize cultivars in Jacaltenango was recorded by Raymond Stadelman [23]. Gathering information initially only in Todos Santos, Stadelman soon realized that in neighbouring villages maize diversity was more abundant - perhaps being informed by Todos Santos maize traders who travelled across the region [25]. Subsequently, Stadelman visited most towns of the region to record data on maize cultivars and maize cultivation. For Jacaltenango he gives 23 names and their main characteristics. Stadelman's lack of reference to LaFarge and Byers' earlier publication, and some discrepancies between the two studies in spelling and interpretation suggest that the two farmer cultivar lists are independent.

In the following sections, Jacaltec cultivar names in the native language will be written in bold, and cultivar names in Spanish will be capitalized. The article follows the modern spelling rules for cultivar names. The unique number between brackets that follows each cultivar name should make comparisons possible, in spite of spelling differences.

## Research question and methods

The main question this research attempts to answer is: Which changes in maize cultivar knowledge occurred during the twentieth century in Jacaltenango? Changes might include both loss of knowledge, and the acquisition of knowledge about new or newly introduced cultivars. It will be attempted to answer this question by using the cultivar lists from 1927 and 1937 as a baseline, to be compared with interview data collected in 2004.

During the last quarter of 2004, a field assistant from Jacaltenango interviewed 40 male farmers in the township capital (*cabecera*) and eight other communities (*aldeas*) in of Jacaltenango (Figure 1; Table 1). Male farmers are generally more knowledgeable on maize diversity in this area [4]. This is probably due to the gendered labour division; men are generally responsible for maize cultivation. Care was taken to include both older and younger informants in the sample for all communities. The communities were chosen to reflect the altitudinal and social variation of the township area. Jacaltenango has three native social segments, called Jacaltenango, San Andrés, and San Marcos, and several foreign segments [33]. As shown in Table 1, the survey covers all three native segments, and several foreign ones.

**Table 1:** Sampled settlements in Jacaltenango (survey in 2004)

Settlement name	Ethnic composition of the settlement (names of 'segments' taken from [33])	Altitude (masl)	Number of interviews
Inchewex	Jacaltenango	900	5
San Andrés Huista	San Andrés	1300	5
Jacaltenango (head town)	Jacaltenango	1400	5
San Marcos Huista	San Marcos	1450	5
Witzobal	San Miguel, Todos Santos, Concepción (all foreign)	1850	5
Cheya	San Miguel (foreign)	1900	5
Acomá	No data	2100	3
El Mul	Foreign	2300	4
Paya	San Miguel, Todos Santos, Concepción (all foreign)	2600	3

The available information was processed following five steps. First, the quality of the baseline data was assessed. Then, the commensurability between the baseline and survey data was evaluated. Having established this, continuity and losses of cultivar knowledge were documented and analyzed. Also, the spatial and social distribution of this knowledge was subjected to further analysis. New cultivars in the area were also documented. The remainder of this section details the methods used for each of these steps.

### Quality of the baseline data

The unique historical data available for Jacaltenango (two independent cultivar lists) permit a limited assessment of the consistency of the classification of maize cultivars by farmers in the past. If a cultivar classification system is fully consistent, the criteria farmers use to assign cultivar names to seed lots should be the same for all farmers. This measure of consistency can be used to compare the reported characteristics of the cultivars recorded by ethnographers in 1927 and 1937, to test the value of cultivar naming in terms of phenotypic diversity. Only if some minimal degree of consistency can be shown, the cultivar names have value to trace diachronic change. The meaning of the cultivar names might contain additional information about the link with biological categories of diversity. The question whether the two cultivar lists give a complete representation of the cultivars that were present in Jacaltenango also needs discussion.

### Commensurability of the baseline and 2004 survey data

If it can be demonstrated that cultivar classification in the first half of the twentieth century is consistent between farmers (previous section), in order to establish meaningful comparisons between two moments, it is also necessary to examine the consistency of cultivar naming over time. The need to establish the stability of cultivar name meanings between 1927/37 and 2004 was foreseen in the interview protocol. In each interview, first, a cultivar name recorded by the 1927/37 studies was mentioned and the farmer was asked if he knew this cultivar. If the answer was affirmative, the farmer was asked what characterized this cultivar. This was asked in relation to (1) adaptation to environment (cold, temperate, hot), (2) grain colour (white, yellow, black, spotted, other), and (3) planting and harvesting dates (given in dates, from which the growing cycle was calculated). This was repeated for all cultivars given in the historical cultivar lists. All three cultivar attributes are available for 1927/37 and these data were used for a comparison to test the consistency of cultivar definitions over time.

### Perceived continuity and losses of cultivars

In the interview, for each historical cultivar known to the farmer, the question was asked whether the known cultivar was still grown (answer: yes/no). The informant was also asked to free-list cultivars that had become rare or had disappeared in his opinion. When the interviewed farmer indicated a cultivar, open questions were asked about the causes of disappearance or rareness.

The answers to the first question were analyzed using methods from 'Consensus Theory' to determine probabilities of presence/absence of each cultivar [37]. The used method employs a measure of informant competence to calculate the probabilities that a certain outcome is true. Informant competence is defined as 'the probability that an informant *knows *the answer'. This definition implies a correction for guessing, which might produce correct answers, while the informant does not know the answer. The theory takes the overall closeness of a particular informant to the other informants as a measure of informant competency. This assumes that consensus between informants is related to the phenomena under study.

The chosen design in the present study deviates in one important aspect from the method proposed by Romney et al. [37]. Throughout the interview, informants had the possibility to indicate they did not know a certain cultivar at all, or did not know if it was still present in the community (leading to missing observations on cultivar presence). Data with missing values are not suited for the analysis proposed by Consensus Theory [38]. A proximate method was taken instead. To calculate agreement between informants, the number of cultivars on which each pair of informant agreed with respect to its absence or presence in the community was divided by the total number of cultivars they both gave a value for present or absent. This leads to a bias: the presence/absence opinions about well known cultivars is taken into account many more times than those for badly known cultivars in the calculation of informant competencies. Therefore, the analysis assumes that the informants' competency in judging the presence/absence of broadly known cultivars is a predictor for their competency to judge the same for less known cultivars.

### Social and spatial distribution of cultivar knowledge

This research question anticipates the possibility of unequal distributions of farmer knowledge between persons and communities. This issue is important for methodological comparisons with regards to sample sizes and distributions. The influence of age on cultivar knowledge will be evaluated, and the influence of environmental conditions and community boundaries. The latter might be important as in the second half of the twentieth century, communities in Jacaltenango tended to become more socially isolated [33].

### Knowledge of new cultivars

Another aspect of knowledge about maize diversity and change is the emergence of 'new' cultivars. Through an open question each informant was asked to identify these cultivars together with some defining characteristics (adaptation, grain colour and growing cycle). This question will also allow assessing to what extent the loss of older cultivars and the emergence of new cultivars are part of a single dynamic of cultivar replacement.

## Results

### Quality of the baseline data

To assess the quality of the baseline data, the two cultivar lists from the early twentieth century were compared. Table 2 summarizes the results of each study and attempts to match the cultivar names from each study as much as possible. In some cases one class corresponds to several (sub)classes in the other study.

**Table 2:** Maize cultivars of Jacaltenango according to two independent sources from 1927 and 1937 Spelling according to original. Abbreviations: C = cold; H = hot; T = temperate; W = warm; m = months. Between brackets: identifying numbers of the cultivars. Dashed lines: separation between growing environments following LaFarge & Byers [36] (see C/T/H classification, second column).

LaFarge & Byers in 1927 [36]		Stadelman in 1937 [23]	
	
Name	Characteristics	Name	Characteristics
			
kex-wa' (1)	C, "black tortilla",	q'ex wa' (1)	C, 9 m
	sweet yellow grain	nime' q'ex wa' (2)	C, 9 m, yellow, intermediate
		papa q'ex wa' (3)	C, 9 m
		kokh q'ex wa' (4)	C, 9 m, yellow, intermediate
tciletcuwa' (5)	C, sweet, white or yellow	t|ilit| wa' (5)	C, 6 m
kěx sat (6)	T, "black eyes"	q'ex sat (6)	C, 9 m
qan-ñal (7)	T, white	q'an ñal (7)	W, 8 m
sax-ñal (8)	T, "white ripe ear"	saq ñal (8)	C, 9 m, white, dent
ts'ip sat (9)	T	ts'ib sat (9)	W, 8 m
ts'ip sat sax-ñal (10)	T, "white ripe ear with written grains"	ts'ib sat saq ñal (10)	C, 9 m, spotted, intermediate

ocep cahua (11)	H, three months, moons	o_ep_xau (11)	W, 4 m, yellow, dent
p:au (12)	H	q'an b:au (12)	W, 8 m, yellow,
nimex kan p:au (13)	H, "big yellow ear"		dent
tcimho (14)	H	Chimbo (14)	W, 6 m
tewa' (15)	H, long term	te wa' (15)	W, 9–10 m
		q'an te wa' (16)	W, 9–10 m

ockal tsaiik (17)	H, "sixty days"	-	-
-	-	q'an wa' (18)	C, 6 m
-	-	?amaltin (19)	C, 9 m, spotted, flint
-	-	jex ti' (20)	C, 9 m, yellow, dent
-	-	saq po (21)	W, 8 m
-	-	Cuarentano (22)	W, 4 m
-	-	Tejar (23)	W, 4 m, white, dent
-	-	Pantaleón (24)	W, 6 m
-	-	q'ex t?itam wa' (25)	black, dent

From the table it is evident that the characteristics mentioned for each cultivar are remarkably consistent. Both studies recorded climatic adaptation for all cultivars except one. LaFarge and Byers split the environments in three zones (cold, temperate and hot), while Stadelman splits them in two (cold and warm). For the two extreme environments of LaFarge and Byers' scale, Stadelman's data show full agreement. For the temperate environment of LaFarge and Byer, Stadelman gives two warm and three cold cultivars, an equilibrated mix. Grain colour data are consistent, also for the cultivar names that do not include colour specifications as part of their name. As LaFarge and Byer did not report on growing cycles, comparisons for this aspect are not possible.

Cultivars are not completely distinguishable using the two mentioned characteristics in Table 2 (environmental adaptation and grain colour). For instance, **k'ej wah **(1) and **kok k'ej wah **(4) are both cultivars of cold environments and with yellow kernel colour. There are two possible situations. First, the latter might be a subgroup of the former class. (In this example, the names suggest the latter cultivar is a subtype of a class which bears the first name.) The other possibility is that the cultivars have other differences not reported by either LaFarge and Byers or Stadelman.

Examining cultivar names might add some information on other relevant differences. In addition to information about kernel colour, environmental adaptation and growing cycle, names contain information on geographic origin. The cultivar Pantaleón (24), like the other cultivars bearing Spanish names, was introduced from a coffee farm in Guatemala's southern piedmont area. There is indeed an existing coffee farm that bears the same name [39]. The name 'Xhamaltin' (19) probably refers to a place called San Martín. However, it could not be determined on the basis of names if cultivar names indeed refer to the smallest units in farmer classifications or refer to broader classes in a hierarchy. Perceptions of farmers in 2004 might not reflect those in the first half of the twentieth century. Therefore, all reported maize cultivar names (n = 24) were included in the analysis.

It is evident that Stadelman's list is more comprehensive than LaFarge and Byers's. Stadelman mentions 23 cultivars, while LaFarge and Byers list thirteen. In two instances, Stadelman gives a finer subclassification of a cultivar mentioned by LaFarge and Byers, while only in one case, LaFarge and Byers split a single cultivar mentioned by Stadelman into two minor units. One cultivar, **ockal tsaiik **(17), is mentioned exclusively by LaFarge and Byers, but our 2004 survey revealed that this cultivar name does not refer to maize, but to common bean (*Phaseolus vulgaris *L.). Assuming that (1) no cultivar change occurred between 1927 and 1937, and (2) that all cultivars had an equal chance to be reported, it might be suggested that Stadelman's list approaches completeness, as it includes all cultivars reported by LaFarge and Byers. However, especially the second assumption is likely not entirely realistic. The fact that some cultivars occur only on one of the lists, might be an indication of their relative scarcity. Even so, taken together, the two lists most likely give an adequate and rather complete picture of Jacaltenango's most common maize cultivars between 1927 and 1937.

### Commensurability of the baseline and 2004 survey data

The 2004 survey included questions on climate adaptation, growing season and grain colour. Comparing the answers to these questions with the historical data gives a measure of the stability of the cultivar classification in Jacaltenango during the twentieth century. Table 3 shows the result of the comparison.

**Table 3:** Comparison for cultivar attributes between historical data and 2004 survey. Most frequent answers are given from the survey. Modern spelling was followed. Cases of disagreement are indicated in bold letter type. Abbreviations: C = cold; H = hot; T = temperate; W = warm; LF&B = LaFarge and Byers [36]; S = Stadelman [23].

	Climate adaptation			Grain colour		Maturity (months)	
			
	Survey	S	LF&B	Survey	S + LF&B	Survey	S
Chimbo (14)	**T**	W	H	-	-	3.7	6
Cuarentano (22)	H	W	-	White	-	2.1	4
k'ej sat (6)	**H**	C	T	Black	Black	7.1	9
k'ejti' (20)	C	C	-	Yellow	Yellow	8.0	9
k'ejti' txitam wah (25)	T	-	-	Black	Black	7.6	
k'ej wah (1)	H/T	C	C	Yellow	Yellow	7.5	9
kok k'ej wah (4)	C	C	-	Yellow	Yellow	6.8	9
nimej k'ejwah (2)	H/T	C	-	**Black**	Yellow	7.4	9
nimej q'anb'aw (13)	-	C	-	Yellow	Yellow	4.1	
oxeb' x'ahaw (11)	H	W	H	**White**	Yellow	2.6	4
Pantaleón (24)	H	W	-	White	-	3.8	6
papa k'ejwah (3)	C	C	-	Spotted	-	6.8	9
q'an b'aw (12)	T	W	-	Yellow	Yellow	5.9	8
q'an nhal (7)	T	W	T	**Yellow**	White	6.5	8
q'an tewah (16)	H	W	-	Yellow	-	6.7	9.5
q'an wah (18)	**H**	C	-	Yellow	-	3.2	6
saj nhal (8)	H/T	C	T	**Spotted**	White	6.2	9
saj poh (21)	H	W	-	White	-	4.3	8
Tejar (23)	H	W	-	White	White	3.7	4
tewah (15)	H	W	-	White	-	6.0	9.5
txilitx wah (5)	C	C	C	Yellow	Yellow/White	8.8	6
tz'ib' sat (9)	T	W	T	Spotted	-	4.6	8
tz'ib' sat saj nhal (10)	T	C	T	Spotted	Spotted	6.9	9
xhamaltin (19)	C	C	-	Spotted	Spotted	5.5	9

Climate adaptation data seem inconsistent only in three out of 25 cases. For Chimbo, in the 2004 survey there is consensus among the informants (n = 3) that it grows in temperate environments. LaFarge and Byers classify this cultivar as being grown in a hot environment. However, as boundaries between adjacent environments are somewhat arbitrary, this case of misclassification might not be relevant. For **k'ej sat **(6) informants mention all three environments as valid for this cultivar, but a majority assigns it to the hot environments. Perhaps the cultivar shows a broad adaptation, and spreads out from the temperate environment (as indicated by LaFarge and Byers) to both warmer and colder environments. The most serious case of misclassification is **q'an wah **(18), which is unanimously classified as a cultivar with an adaptation to hot environments (n = 5), while Stadelman reported it was adapted to cold growing environments. However, generally the data are consistent.

The most common answer on colour data disagrees with the historical data in four of the fifteen cases the latter data are available. In three of the four cases of disagreement, currently little consensus exists and the colour mentioned in the historical source is also frequently mentioned (data not shown). In the remaining case, the historical data might be wrong, when it classifies **q'an nhal **(7) as white, as the name of this cultivar includes an element (**q'an**) that means yellow.

**Figure 2:**
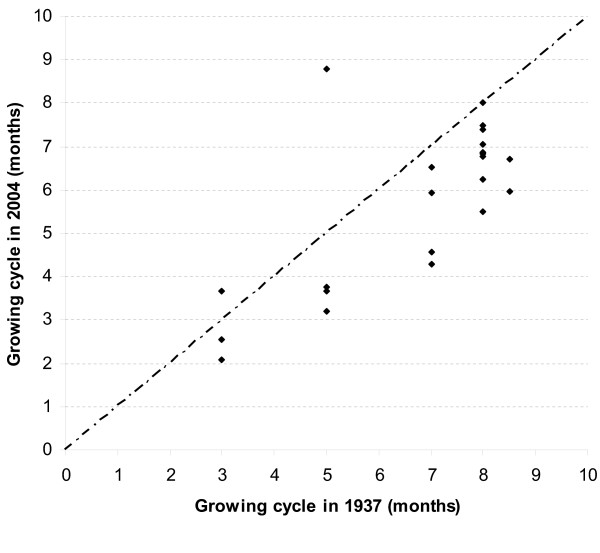
Growing cycle of cultivars compared between data of 1937 (Stadelman [23]) and 2004 (survey). Dashed line indicates a hypothetical 1:1 relationship.

The time difference between planting and harvesting was taken as the length of the growing cycle for each cultivar for both Stadelman's data and the 2004 survey data. There is a significant, positive correlation between the two datasets for growing cycle length (r^2 ^= 0.54; p = 0.0001). There is a systematic change in the cultivars with longer growing seasons; they are generally under the 1:1 line in Figure 2. Stadelman reported planting dates in April for all highland cultivars, while according to the 2004 survey May or June is the norm. Rainfall and soil moisture early in the season might have become more limiting in recent decades. Given that the tendency is present across the whole sample, it does not interfere with cultivar identity. There is, however, one outlier: **txilitxwah **(5). According to Stadelman this cultivar is the only one for cold environments that has such a short growing season (Table 2). This exceptional status might explain the discrepancy; Stadelman or his informants probably made a mistake.

Overall, the consistency between the historical data and the data of the survey is strong enough to conclude that the mentioned cultivars are very likely the same ones in 1927/37 and in 2004. This suggests the data on farmer's knowledge of cultivar occurrence are sufficiently reliable to determine the continuity or disappearance of the cultivars that were present at the beginning of the twentieth century in Jacaltenango.

### Perceived continuity and losses of cultivars

All cultivar names recorded by Stadelman or LaFarge and Byers in the first half of the twentieth century were recognized by some of the informants in 2004 (n = 40), varying from 3 informants for the least known cultivars to 39 for the best known (Table 4). Informants knew 13.7 cultivars on average (57.1%), varying from 7 to 20 (SD = 2.9).

**Table 4:** Cultivar knowledge and opinions on presence/absence (n = 40). Last column calculated using Consensus Theory (Appendix).

	Informants who know the cultivar	Communities in which cultivar is known (n = 9)	Informants who judge presence	Informants who claim continued presence	Informants who claim continued presence as a percentage of all informants who judge presence	Probability of continued presence
nimej k'ejwah (2)	39	9	35	34	97%	>0.99
k'ejwah (1)	38	9	34	34	100%	>0.99
saj nhal (8)	38	9	33	32	97%	>0.99
tz'ib'sat saj nhal (10)	35	9	31	29	94%	>0.99
k'ejsat (6)	32	9	31	27	87%	>0.99
tz'ib'sat (9)	32	9	30	26	87%	>0.99
q'an nhal (7)	31	9	26	26	100%	>0.99
q'an b'aw (12)	31	9	26	21	81%	>0.99
tewah (15)	31	9	27	19	70%	>0.99
kok k'ej wah (4)	30	9	27	24	89%	>0.99
txilitxwah (5)	29	9	25	22	88%	>0.99
oxeb' x'ahaw (11)	27	9	26	25	96%	>0.99
Cuarentano (22)	26	8	23	22	96%	>0.99
k'ejti' txitam wah (25)	25	8	24	23	96%	>0.99
papa k'ejwah (3)	23	9	22	21	95%	>0.99
q'an tewah (16)	19	7	12	9	75%	>0.99
nimej q'anb'aw (2)	18	8	15	13	87%	>0.99
xhamaltin (19)	12	6	10	9	90%	>0.99
saj poh (21)	12	6	11	7	64%	>0.99
q'an wah (18)	6	5	5	4	80%	>0.99
Pantaleón (24)	4	3	2	0	0%	0.07
k'ejti' (20)	3	3	3	3	100%	>0.99
Chimbo (14)	3	2	2	1	50%	0.25
Tejar (23)	3	2	3	0	0%	0.07

For 39 informants cultivar presence/absence judgments are available. According to the Consensus Theory analysis, 77% (30 out of 39) of the informants have a competence of more than 0.8 and 67% (26 out of 39) exceed 0.9. Average competence is 0.85. These high competence numbers indicate that the judgments are generally consistent among different informants. Thus, even though the number of responses for the rare cultivars are low (Table 4), as could have been expected, given the informant competencies calculated on the basis of the whole range of cultivars, a probability of the presence for these cultivars can be calculated.

There is no significant correlation between informant competence and the number of cultivars informants judged, or their perceived Spanish language skills (p < 0.05). Age has a weak negative correlation with competence (r^2 ^= 0.14; p = 0.02). Since most deviations from consensus are related to absence judgements of cultivars, it follows that older informants tend to be slightly more pessimistic about cultivar presence than younger informants. However, older informants also know more cultivars than younger informants, which is a stronger tendency (r^2 ^= 0.24; p < 0.01). Thus, age related differences in knowledge did probably not influence the findings of this study.

In Table 4, counts for cultivar knowledge and opinions of presence and probabilities of presence (following Consensus Theory) are presented. The data show a general agreement between the (perceived) presence of the cultivar by the informants that know the cultivar and the knowledge of the cultivar across the whole population of informants. However, the association is not complete. For instance, **k'ejti' **(20) is known by only three informants, but according to these informants the cultivar is still present. In contrast, Tejar (23), another cultivar known by only three informants, probably ceased to exist in Jacaltenango. Another interesting characteristic is that even the most rare cultivars were always known in at least two communities

Following these findings, three cultivars have disappeared in Jacaltenango during a 70 year period. All three cultivars that were likely lost, have Spanish, not native Popti' names. According to informants, these cultivars were introduced originally from coffee plantations to which Jacalteco workers temporarily migrated in the coffee harvest season to work. Several causes for the disappearance or scarcity of cultivars are mentioned. There is no clear pattern apparent in the causes in relation to certain cultivars; most causes apply to all. The most important reason is the yield disadvantage of the traditional cultivars against the introduced cultivars. With the same fertilization levels, traditional cultivars yield less. They also grow taller and are more prone to lodging (the bending over and falling of plants). The introduction of industrial fertilizers in the 1960s [40] has accentuated this problem, as cultivars developed even more biomass. The higher disease susceptibility of the cultivars Chimbo (14) and Pantaleón (24) was also mentioned as a reason for their disappearance. Another reason the informants mentioned was climate change. According to some informants the growing environment has become warmer and drier. Land use change (more coffee) was also mentioned (this is also a primary cause for the high teosinte extinction risk, G. Wilkes, pers. comm., December 2004).

### Social and spatial distribution of cultivar knowledge

In Table 5, the distribution of cultivar knowledge over communities, informants and cultivar adaptation groups is given. A single-factor analysis of variance for differences in cultivar knowledge among communities shows that the community means are not equal (p = 0.02) (mean age of informants was not significantly different between different communities).

**Table 5:** Social and spatial distribution of cultivar knowledge per adaptation group The assignment of cultivars to adaptation groups is based on the results of the 2004 survey (see Table 3).

Number of cultivars known per informant	Inchewex	San Andrés Huista	Jacaltenango	San Marcos Huista	Witzobal	Cheya	Acoma	El Mul	Paya	Mean all informants	Total
Hot	2.4	2.2	1.6	3.0	2.0	3.2	2.7	3.25	0.3	2.3	8
Hot and temperate	2.8	3.0	2.8	2.6	2.4	2.6	2.7	2.75	3.0	2.7	3
Temperate	3.8	2.8	2.4	2.6	2.0	3.6	3.7	4.25	2.3	3.1	6
Cold	5.8	5.2	4.4	6.2	5.8	6.4	5.7	5.75	4.7	5.5	7
All environments*	14.8	13.2	11.2	14.4	12.2	15.8	14.6	16	10.3	13.7	24

Total of cultivars known per community	Inchewex	San Andrés Huista	Jacaltenango	San Marcos Huista	Witzobal	Cheya	Acoma	El Mul	Paya	Mean	Total

Hot	4	5	3	7	4	7	4	5	1	4.4	8
Hot and temperate	3	3	3	3	3	3	3	3	3	3.0	3
Temperate	5	5	5	5	5	6	5	5	4	5.0	6
Cold	7	7	7	7	7	7	7	7	7	7.0	7
All environments	19	20	18	22	19	23	19	20	15	19.4	24

*The means are not equal among communities (ANOVA; p = 0.02)

Knowledge of cultivars grown in cold and temperate environments is roughly stable across communities. The most significant differences exist in knowledge of the cultivars in the hot growing environment. The township capital of Jacaltenango itself is ranking the second lowest in number of cultivars per informant and the total number of cultivars known. Together, the five informants from the township capital only knew three out of eight cultivars adapted to hot environments. The only community that scored worse was Paya, where only three informants were interviewed and which is the community that is most remote from the low area in distance and altitude of all sampled communities (Figure 1).

These observations strongly suggest that spreading the interview sample over several communities might have enhanced the research design. It also suggests that relying on interviews in the township capital alone would have led to serious underestimations of farmer knowledge of historical cultivars in Jacaltenango. This is an important point about method and will be taken up in the discussion.

### Knowledge of new cultivars

Table 6 gives the names that were mentioned for cultivars that were not included in the historical data. At least a large majority of this list of cultivars is introduced during recent decades, and from the 2004 survey data no additional historical cultivars (being in Jacaltenango for more than 70 years) could be added.

**Table 6:** Introduced cultivars in Jacaltenango

Cultivar	Grain colour (most common answer)	Climate adaptation (all answers)	Growing season (mean, months)	Number of informants who mention this cultivar
Reina	Yellow	Hot/temperate	5.1	13
Crema	White	Hot/temperate	5.3	8
ICTA	White	Hot/temperate	4.5	7
Grano de oro	Yellow	Hot/temperate	4.3	6
saj sat	White	Hot/temperate	5.0	4
Conejo	Yellow	Hot	2.7	3
Tuxpeño	White	Hot/Temperate	4.0	3
Lucas	Yellow	Hot/Temperate	4.3	2
Taxa	White/Yellow	Hot	5.0	2
Siete hojas	White	Hot	3.0	2
Manuel Juan	White	Hot	5.0	2
Juncanero	White	Temperate	5	1
Mapalu	White	Hot	4	1
Americano	White	Hot	4	1
yixim chik	Yellow	Temperate	4	1
saj k'o ixim	White	Temperate	6	1
kej k'o ixim	Black	Temperate	6	1
Cinco pies	White	Hot	4	1
Rocamey	White	Hot	4	1
Tropical	White	Hot	4	1
caj chil	Yellow	Hot	3	1
Super enano	White	Hot	4	1
Sintalapa	White	Hot	5	1
Máquina	No data	No data	No data	No data

More than half of the introduced cultivars are grown only in hot environments, and only four are grown in temperate climates alone. This tendency corresponds to the pattern of cultivar loss: the lost cultivars were adapted to warm environments. Among the grain colours, white dominates. This is generally the commercial grain in Guatemala, whereas most yellow grain is for home consumption. Most of the new cultivars are fast growers (average: 4.4 months). A short growing cycle, lower plant stature, and a higher yield was indicated as an important reason for their introduction.

The informants reported that the introduced maize was coming from various geographical sources, which are partly reflected in the cultivar names. Maize seed comes from the commercial maize growing areas of the Pacific coast (reflected in the cultivar name 'Máquina', which refers to an important maize growing area of the Pacific coast, called La Máquina), the national agricultural institute (ICTA), and Mexico (Tuxpeño, and probably others). The influx from planting materials from Mexico might be related to the return of refugees who fled to Mexico during the political violence of the 1980s. "Rocamey" in Table 6 probably refers to Rocamex, a variety introduced in the 1960s in broad areas of Central America, which was originally bred at the Mexican Agricultural Program of the Rockefeller Foundation in Mexico. The names also contain information on the person who introduced it (Lucas, Manuel Juan).

## Discussion

### Cultivar names

On basis of the criteria applied in this study, cultivar names were generally consistently related to biological characteristics. Cultivar characteristics between the two historical data sources showed close correspondence. The same was true for the comparison between the historical data and the data for the 2004 survey. Also, in the few cases a disagreement was detected, often a reasonable explication was available. This suggests that cultivar names generally refer to the same units of maize diversity, as distinguished by farmers.

For the first half of the twentieth century and also for 2004, classification of maize diversity imply more than phenotypic categories. It includes additional information about the geographic origin, and in the case of more recently introduced seeds, the person responsible for the introduction. This suggests that in some cases, cultivars might be distinguished not on the basis of visible characters or use, but their history. It seems that this is increasingly so, because the new cultivar names reflect a plethora of incoming diversity, and the names suggest that categorization is no longer mainly related to phenotypic categories.

It may be concluded that farmer cultivar names partly reflect the use and history of seeds, but that for the cultivars included in the baseline data, phenotypic differences played a relatively important role in classification and naming. Morphological and genetic studies are needed to further examine the biological meaning of cultivar names in Jacaltenango and other parts of the western highlands of Guatemala. Meanwhile, it may be assumed that cultivar differences are relevant biologically.

#### Cultivar change in Jacaltenango

The findings suggest that a small loss of historical maize cultivars occurred. Mainly factors related to production shape the way in which maize cultivar change occurs in Jacaltenango. Motivations for change are related to cultivar characteristics related to their production ecology, specifically plant height, growing cycle and disease problems. Broader underlying causes included a perceived climate change and the introduction of fertilizers. Climate change towards a lower annual precipitation and higher annual temperatures over the last century has indeed been documented for the region [41].

Loss of cultivars is localised in the lower areas of the township and limited to those cultivars which had been introduced before 1937 from coffee farms outside the community. Since the original source of the replaced historical cultivars is regional, they are probably not of unique value. A regional assessment of cultivar loss is necessary to determine if this phenomenon is general. However, the production problems the lost cultivars reportedly had, suggest farmers do not regret these losses.

The prevalence of crop ecological factors in cultivar loss suggests that the political violence of the 1980s has had led to little or no loss of cultivars. Thus, our study disproves Steinberg and Taylor's [4] suggestion that political violence in the 1980s would have led to a sweeping loss of maize cultivars. In spite of many deaths and massive migration, the continued residence of some groups in the village even at the heights of violence (civil patrols, for instance), the short absence of others, and the possible exchange and recuperation of seeds, apparently helped to conserve farmer cultivars. It should be noted that a detailed study of the impact of the genocide and violence in Rwanda reported little *absolute *loss of bean, potato and sorghum genetic diversity, although it noted problems with accessing diversity by particular farmers and (in the case of potatoes) acquiring sufficient volumes of planting materials [42].

The fact that none of the informants knew all historical cultivars (nor all informants from each community together), implies that very likely a change in the relative abundance of the historical cultivars occurred. (However, see below for the possibly complicating effect of changed distributions of knowledge.) This effect is strongest for the cultivars adapted to warm environments. This is an important finding, which may be related to another aspect of cultivar change: the introduction of seeds from other areas into Jacaltenango.

The many new cultivars mentioned by informants are largely confined to the lower areas of Jacaltenango. Several foreign cultivars have been introduced to temperate parts of the townships, but less than to warm environments. No new cultivar for cold environments was reported. This difference in relative openness of low and high parts of the landscape for cultivars from outside reflects a broader trend in maize biogeography. Genetic studies based on maize materials available before the introduction of improved varieties, have observed increased genetic isolation with increased altitude [13]. This suggests that rather stable, ecological constraints to seed and cultivar exchange underlie the differences between high and low areas.

Extracommunal seed sources changed over the twentieth century. Before 1937, the sources of cultivars outside Jacaltenango included mostly the coffee farms in the southern piedmont areas. In the last decades, the focus shifted towards the formal seed sector (ICTA, agricultural input shops), the commercial maize growing areas that were developed on the Pacific Coast, towards Mexico. Cross-border contacts increased as many people fled to Mexico following political violence in the 1980s. Thus, in this way political violence did influence maize diversity in Jacaltenango.

The new cultivars in Jacaltenango are mostly recycled seed lots that stem from modern varieties. Their reported advantages (lower plant stature, shorter growing cycle, higher yields) indicate that the motivations for cultivar change are crop ecological. The production problems that motivate cultivar change are also present in the higher areas of Jacaltenango. In the cold environments no change was observed. It might be true that poor access to foreign cultivars adapted to this area constrains cultivar change in the higher parts. Further examination of this possibility is needed.

Three findings suggest that the loss or rareness of older cultivars and the introduction of new cultivars in Jacaltenango might be part of one coordinated long-term trend of cultivar replacement. First, cultivar losses and introductions take place in the same growing environment, the lower parts of Jacaltenango. Second, for both processes, similar ecological motivations are mentioned by farmers in the area. Third, in the interviews, farmers often made direct comparisons between the older cultivars on the one hand, and the newly introduced cultivars on the other hand, especially in terms of yield. In this case there are strong indications that replacement may be an important aspect of cultivar change in the lower areas. However, since many of the cultivars reported in 1927/37 are still present, apparently households have certain reasons to conserve them. The present study was not able to uncover these reasons.

### Methodological comparisons with an earlier study

In 2001, geographers Michael K. Steinberg and Matthew Taylor did a field study in highland Guatemala with the hypothesis that political violence would have caused major maize cultivar loss [4]. Their study comprised six townships in the department of Huehuetenango, including Jacaltenango. The authors used Stadelman's [23] report as baseline data, and interviewed ten persons from each township capital to compare their knowledge to Stadelman's list. Steinberg and Taylor conclude that cultivar knowledge has diminished severely in this area since the early twentieth century, from 30 to 13 cultivars. For Jacaltenango, Steinberg and Taylor found that cultivar knowledge diminished from eight to three cultivars (a loss of 62.5%). Steinberg and Taylor imply that Stadelman reported only eight cultivars for Jacaltenango, while the present study derives 23 cultivars from Stadelman's text (Table 1). Steinberg and Taylor used an incomplete table from Stadelman's report, that referred to the ears he collected (Table VII in Stadelman [23], M.K. Steinberg, pers. comm., 24-06-2005). Although Steinberg and Taylor emphasise the preliminary character of their study, since the present study estimates that cultivar loss in Jacaltenango was considerably lower (around 13%), a detailed comparison between the methodologies of the two studies is warranted.

Steinberg and Taylor modelled their sampling method on the one used by the ethnographers of the first half of the twentieth century. So given equal methods, if farmers reported less cultivars to Steinberg and Taylor than to Stadelman in several townships, this would suggest change occurred. However, the method employed by Steinberg and Taylor does not provide information about the certainty of this outcome.

The method of Steinberg and Taylor estimates cultivar loss directly from the total number of cultivars known by a small number of farmers in each township. The present study shows the least known cultivars include those which are judged to be no longer present. This would support Steinberg and Taylor's method in general, but misleads in the case of lesser known cultivars that continue to exist. In Jacaltenango, at least one cultivar was as little known as the cultivars that were deemed to have disappeared, but was thought to be still present.

A more important issue is, however, that in Steinberg and Taylor's methodology no judgment can be made about whether the number of interviews is sufficient to have a certain degree of certainty about the outcomes. More intensive sampling will tend to increase the number of cultivars known by informants, thus changing the outcome. A related problem is where to draw the boundary between present and absent cultivars when in fact all cultivars are still remembered (as is the case of the present study). Steinberg and Taylor's overestimation of cultivar loss (or their suggestion in this direction) is a direct consequence of a lack of checks in their method.

One possible interpretation of the study of Steinberg and Taylor is that it provides information on the *relative *abundance of historical cultivars in comparison with the past. However, it should be indicated that this would assume that the township capital in 1937 and 2004 are equivalent units of analysis. This assumption can be discussed in the light of the findings obtained with the methodology presented in this article, which give some clues about the current spatial distribution of cultivar knowledge (next section).

### Social and spatial distribution of cultivar knowledge

Several findings of the present study point to an unequal social distribution of maize cultivar knowledge in 2004. First, informants from some communities knew less historical cultivars than informants from other communities. Informants in Jacaltenango in this study proved to be some of the least knowledgeable on maize cultivars. The poor knowledge of farmers in the capital town might have led to the underestimation of farmer knowledge in Steinberg and Taylor's study, whose informants were encountered in the capital towns only. Second, the incoming cultivars show an extraordinary number of cultivar names, many being mentioned by one informant only. It seems as if the numerous cultivar introductions outstrip the capacity of seed and knowledge exchange in Jacaltenango.

Stadelman's [23] data indicate that a knowledgeable male adult living in the township capital of Jacaltenango might know many of the less abundant cultivars of the area. The present study suggests that in 2004 the same was true for many informants from communities in Jacaltenango, but not for all informants, including those from the township capital. This finding might reflect a change in the distribution of maize between the head town and the other communities, which have a more rural character, and perhaps between the rural communities as well.

Broader socio-economic trends might explain a possible change in the social distribution of maize knowledge. In the first half of the twentieth century, maize was more important for the monetary economy than in 2004, and a main node in this economy was the head town. Now, maize has a minor role, while other crops (especially coffee) and other occupations have become more prominent economically. Economic change might have diverged interest into other issues than maize diversity. Another possible explanation is a decrease in knowledge transmission. Intergenerational knowledge transmission might still underpin the social memory about disappeared cultivars that was observed in this study. However, the growing population of Jacaltenango, increasing social isolation and independence between communities and their increasing regional and national orientations [33,43] might have added to a fragmentation of traditional agricultural knowledge systems in rural Guatemala. Also, the political violence of the past might have reduced the trust and solidarity that underpins seed and knowledge exchange [44].

## Conclusion

This study has described the application of a methodology to examine change in farmer knowledge of cultivars. It has demonstrated that sensible results can be obtained with the used methodology, which might have interesting implications for biological change as well. By taking a spatially stratified sample in an area of exceptional cultivar knowledge, rich ecological diversity and presumably maize biodiversity, it produces information that might be impossible to obtain in a regional investigation, but contains insights that possibly apply to a much larger area than the extent of this study.

This article has shown that maize cultivars names identified three generations earlier in a Guatemalan highland township are still present in the social memory. Relative certainty existed about certain trends of cultivar change in the township, which in broad terms correspond to the same perceptions of biological diversity, where their consistency could be tested. Consensus existed about the disappearance of a small number of cultivars adapted to warm growing environments (below 1,500 masl) due to problems related to crop production. This was also the area where cultivar introductions from other areas concentrated. Ecological factors are reportedly important in cultivar change, perhaps contributing to a slow replacement of the older cultivars with new ones. Given the importance of ecological factors, these insights might prove to apply to broader areas with similar ecologies. One question that merits special attention are the production problems of the high environments in the study area, which are perhaps as serious as the problems in the low environments, but do not seem to have obvious (seed-based) solutions.

The reported research has generated various insights in the role of social factors in cultivar change. Political violence did evidently not cause an observable absolute loss of cultivars in the study area, contrary to the expectations raised by earlier research. On the other hand, it was observed that the regional social connections that underpin cultivar introductions changed in geographical focus over the twentieth century. As these changes are part of broad socio-economic trends they might affect other parts of the region as well. Also, several findings suggest a change in the social and spatial distribution of cultivar knowledge within the township during the twentieth century. This paper substantiates that (changing) knowledge distributions potentially constitute an important issue for methodology and interpretation in research on change in cultivar knowledge.

## Appendix: Consensus analysis

For the consensus analysis, the proportion of presence/absence agreement was calculated for each pair of informants for the cultivars known in common only. This was corrected for possible agreement due to guessing, following Romney et al. [37]. The resulting matrix was loaded as a correlation matrix into SAS 9.1 for Windows [45], and analyzed using the principal components method of the Factor procedure and a Varimax rotation. The first factor solution corresponded to 79.8 % of the variance, and the second and third corresponded to 10.8 % and 7.3 % respectively. The high value for the first factor compared to the next ones partially confirms the suitability of consensus theory for these data [37]. Factor loadings for the first factor solution included one negative value (-0.07). Since negative knowledge or sabotage seems unlikely, this indicates that the correction for guessing may lead to conservative (underestimated) informant competence values. Constraining presence judgements to known cultivars perhaps filters out much guessing already. The first-factor loadings for each informant were used as competence values. From these, the probability of presence for each cultivar was calculated, following Romney et al. [37].
